# *Bacillus subtilis* MraY in detergent-free system of nanodiscs wrapped by styrene-maleic acid copolymers

**DOI:** 10.1371/journal.pone.0206692

**Published:** 2018-11-05

**Authors:** Yao Liu, Elisabete C. C. M. Moura, Jonas M. Dörr, Stefan Scheidelaar, Michal Heger, Maarten R. Egmond, J. Antoinette Killian, Tamimount Mohammadi, Eefjan Breukink

**Affiliations:** 1 Department of Membrane Biochemistry and Biophysics, Institute of Biomembranes, Utrecht University, Utrecht, the Netherlands; 2 Department of Experimental Surgery, Academic Medical Center, University of Amsterdam, Amsterdam, the Netherlands; University of Cambridge, UNITED KINGDOM

## Abstract

As an integral membrane protein, purification and characterization of phospho-*N*- acetylmuramyl- pentapeptide translocase MraY have proven difficult. Low yield and concerns of retaining stability and activity after detergent solubilization have hampered the structure-function analysis. The recently developed detergent-free styrene-maleic acid (SMA) co-polymer system offers an alternative approach that may overcome these disadvantages. In this study, we used the detergent free system to purify MraY from *Bacillus subtilis*. This allowed efficient extraction of MraY that was heterologously produced in *Escherichia coli* membranes into SMA-wrapped nanodiscs. The purified MraY embedded in these nanodiscs (SMA-MraY) was comparable to the micellar MraY extracted with a conventional detergent (DDM) with regard to the yield and the purity of the recombinant protein but required significantly less time. The predominantly alpha-helical secondary structure of the protein in SMA-wrapped nanodiscs was also more stable against heat denaturation compared to the micellar protein. Thus, this detergent-free system is amenable to extract MraY efficiently and effectively while maintaining the biophysical properties of the protein. However, the apparent activity of the SMA-MraY was reduced compared to that of the detergent-solubilized protein. The present data indicates that this is caused by a lower accessibility of the enzyme in SMA-wrapped nanodiscs towards its polyisoprenoid substrate.

## Introduction

Membrane proteins play pivotal role in the biological system. A major obstacle of studying integral membrane proteins is the extraction and purification of the membrane proteins in their active form. The use of detergents has been widely practiced but this also leads to disruption of the native lipid bilayer environment, which can be important for maintaining the functional properties of the studied membrane protein. Recently, the development of a styrene-maleic acid (SMA) copolymer [1] as alternative of detergent has shed light on membrane protein research. This amphipathic copolymer is able to spontaneously self-insert and extract membrane patches with the membrane protein embedded in its native lipid environment [1–5]. This system doesn’t require an intermediate detergent solubilization step and does not disrupt the native lipid bilayer. This strategy has been successfully implemented to purify several membrane proteins from *E*. *coli*, plants, and mammalian cells that are otherwise unstable or lose functionality after extraction [1–4, 6, 7]. In the present study, we examine the use of SMA copolymer in extracting an important bacterial membrane protein, MraY.

Most bacteria are surrounded by a protective layer known as the peptidoglycan layer of the cell wall. To build this distinctive and essential structure [8, 9], bacteria have to undergo a multistep process that involves several enzymes, including MraY. In brief, the biosynthetic pathway of peptidoglycan starts at the cytoplasm where soluble precursors are synthesized through the successive action of Mur enzymes, resulting in the precursor: UDP-*N*-acetyl-muramyl-pentapeptide (UDP-MurNAc-pentapeptide). The MurNAc-pentapeptide moiety is then attached to undecaprenyl phosphate at the cytosolic side of the membrane by MraY, thus forming Lipid I. Subsequently, the glycosyltransferase MurG catalyzes the formation of Lipid II by coupling GlcNAc (*N*-acetyl-glucosamine)to Lipid I. Lipid II is then translocated across the cytoplasmic membrane to the periplasmic space where it is used to synthesize mature peptidoglycan after processing by penicillin-binding proteins [10–12].

Owing to its essential role [10–12] in the peptidoglycan biosynthesis described above, MraY has been the subject of many studies attempting to identify inhibitors [13, 14]. Exploration of MraY as a target for identifying inhibitors such as in high-throughput screening assays requires sufficient amounts of purified protein. As an integral membrane protein, MraY spans the cytoplasmic membrane of bacteria with 10 transmembrane helices [15]. The importance of the native lipid environment in preserving the function and stability of MraY has been demonstrated in different studies [15–19]. Among these, the study published recently by Henrich et al. [18] reported that MraY enzymes originating from Gram-negative bacteria required the presence of negatively charged lipids in order to maintain their stability and enzymatic activity. In general, biochemical analyses and studies addressing its structure-function are limited [20–23]. In particular, the catalytic mechanism of this protein still remained unclear until recently [18,20]

In the present study, we adopted the SMA copolymer method to extract MraY of *Bacillus subtilis* origin produced in *E*. *coli* in its active form. We compared the performance of this SMA system to that of the conventional detergent system, i.e., using *n*-dodecyl-ß-D-maltoside (DDM). Both systems show comparable results with regard to purity and yield. MraY encapsulated in the nanodiscs was more stable against heat or protease degradation. However, it presented reduced apparent activity, which is likely due to reduced access of the polyprenoid substrate towards the enzyme based on the results of a series of activity/kinetic tests.

## Methods

### Materials

Unless stated otherwise all chemicals used were purchased from Sigma Aldrich (Saint Louis, MO, USA); all DNA ladders, restriction enzymes and their buffers were purchased from Thermo Fisher Scientific (Waltham, MA, USA).

Primers for DNA amplification and sequencing were synthesized by Biolegio (Nijmegen, the Netherlands) or Integrated DNA technologies (Coralville, IA, USA). Sequencing services for all DNA constructs were provided by Macrogen (Amsterdam, the Netherlands). pGEM-T Easy cloning system I was purchased from Promega (Fitchburg, WI, USA). pET28a vector was purchased from Novagen (Darmstadt, Germany). EDTA-free protease inhibitor cocktail tablet was obtained from Roche diagnostics (Basel, Switzerland). *n*-dodecyl-β-D-maltopyranoside (DDM) was obtained from Anatrace (Maumee, OH, USA). Precision Plus Protein standards were purchased from Bio-Rad Laboratories, Inc. (Hercules, CA, USA). Isopropyl-β-D-thiogalacto- pyranoside (IPTG) was purchased from Thermo Fisher Scientific. Δ*sly*D BL21 (DE3) *E*. *coli* strain was a kind gift from Prof. Ry Young (Dept. Biochemistry and Biophysics, Texas A&M University, USA).

### Heterologous production of MraY in *E*. *coli*

The gene encoding MraY was amplified from *Bacillus subtilis* 168 using the primer pair: BamHI-BY: 5’-ggatccatgcttgagcaagtcattcgtttac-3’, and BY-HindIII: 5’-aagcttttataaccacacc- tcg-3’. The restriction sites are underlined. The PCR product was digested with BamHI and HindIII and ligated into the same sites of the pET28a vector, which carries an N-terminal His_6_ affinity tag. The resulted expression plasmid was named pET28aBsY.

To avoid additional steps to remove SlyD, a known contamination of his-tagged protein purification [24], we routinely used a SlyD deficient strain of *E*. *coli*, Δ*slyD* BL21(DE3) that was described previously [25, 26] for production of MraY. pET28aBsY was freshly transformed into competent Δ*slyD* BL21 (DE3) [25, 26] cells and subsequently inoculated into 5 mL of Luria Broth (1% trypton, 0.5% yeast extract, and 1% NaCl) supplemented with kanamycin (50 μg/mL) for overnight growth at 37°C. This pre-culture was diluted 1:100 in pre-warmed (37°C) Terrific Broth (1.2% tryptone, 2.4% yeast extract, 0.4% glycerol, 17 mM KH_2_PO_4_ and 72 mM K_2_HPO_4_) supplemented with kanamycin (50 μg/mL). Growth was continued at 37°C up to an OD_600_ of about 0.5. Protein expression was then induced by adding IPTG to a final concentration of 100 μM. Subsequent culturing was continued at 22°C for an additional 4 h.

### Membrane solubilization with n-dodecyl-ß-D- maltoside detergent

Cells were then collected by centrifugation at 4,000 g for 30 min at 4°C. The pellets were washed in 10 mL /1 L culture buffer consisting of 25 mM Tris-HCl, pH 7.6, 150 mM NaCl, and 10% glycerol (Buffer A) supplemented with 1 mM phenylmethylsulfonyl fluoride (PMSF) and protease inhibitor tablet (complete EDTA-free, Roche), 20 μM DNase, 20 μM RNase and 0.25 mg/mL lysozyme. This suspension was subjected to probe sonication (200 Watt, 5–10 times, 10 s bursts), while keeping cells cool in an ice bath. The suspension was then centrifuged at 12,000 g for 10 min at 4°C to remove the unbroken cells. Membrane pellets were then prepared by centrifugation at 206,000 g for 45 minutes at 4°C. The pellet (containing membranes and associated proteins) was resuspended in Buffer A (10 mL/5 L culture). Membranes were solubilized by addition of DDM to a final concentration of 1%. The mixture was incubated at 4°C for 1.5 h with agitation. After ultracentrifugation (206,000 g for 45 min at 4°C), the first supernatant was recovered. The remaining pellet was subjected to another round of solubilization. The supernatants were pooled and incubated with Ni-NTA-agarose beads (pre-equilibrated with 20 mM imidazole in Buffer A) at 4°C with mixing for 2.5 h.

The resin was transferred to a gravity-flow column and extensively washed with Buffer A containing 50 mM imidazole. The protein was then eluted with Buffer A containing 250 mM and 500 mM imidazole. Purity of the protein was assessed by LDS-PAGE (adapted from NuPAGE from Life Technologies using 4× LDS sample buffer), and Image J analysis followed by staining with either silver nitrate or Coomassie dye. The protein concentration of the purified MraY was approximated by densitometry using bovine serum albumin (BSA) as standard.

### Membrane solubilization with styrene maleic acid copolymer

Xiran SZ30010, a styrene-maleic anhydride copolymer with a molar styrene-to-maleic anhydride ratio of 2.3:1 and an average molecular weight of 9.5 kDa, was a kind gift of Polyscope (the Netherlands). It was converted to styrene-maleic acid (SMA) as previously described [27]. Briefly, the polymer was hydrolyzed in 1 M KOH under reflux for 2 h while heated to 100°C. This was followed by precipitation of SMA by the addition of HCl and then 6 washing steps with 100 mM HCl to remove K^+^. The mixture was lyophilized to obtain SMA dry powder, which was stored at room temperature until further use. A 6% (w/v) SMA solution was prepared by dissolving the SMA powder in 50 mM Tris solution upon heating with gradual addition of NaOH until the pH reached 7.8.

All following steps were carried out at 4 ˚C to solubilize the membranes. *E*. *coli* cell pellets harboring MraY were collected from 5 L culture, washed, and resuspended in 2-fold concentrated Buffer A. The 6% SMA solution was added 1/1 (v/v) to the cell suspension and the mix was subsequently passed through the cell disruptor (Constant systems, Daventry, UK) at a pressure of 2.10 kbar. Unbroken cells were removed by centrifugation at 12,000 g for 10 min at 4°C. To allow complete solubilization, the supernatant was incubated with agitation for another 1.5 h at 25°C. After the pH was adjusted to 8.0, the supernatant was incubated overnight at 4˚C with Ni-NTA-agarose beads pre-equilibrated with Buffer A supplemented with 20 mM imidazole. The protein was further purified and eluted following the same procedures as described in the previous section.

### Transmission electron microscopy

SMA-MraY at a concentration of 0.28 μM was mixed with 0.5 μM Ni-NTA-nanogold particles of 5 nm (Nanoprobes Inc., Yaphank, NY, USA) in Buffer A (pH 7.6, total volume = 20 μL) and incubated overnight at 4°C. The mix was transferred to a glow-discharged copper grid coated with Quantifoil polymer film and absorbed for 2 min. Excess sample (10 μL) was removed by filter paper. The grid was then stained with ammonium molybdate solution (2%, 5 μL) for 45 s and briefly dried with filter paper. The samples were observed under a transmission electron microscope (Tecnai 12, Philips) operating at an acceleration voltage of 120 kV.

### Circular dichroism spectroscopy

The purified protein fractions, 140 nM DDM-MraY and 70 nM SMA-MraY, were dialyzed using a 1 kDa cut-off dialysis membrane against a CD-compatible buffer containing 15 mM NaCl and 1 mM phosphate (pH 7.6). Far-UV CD experiments were performed on a J-810 spectropolarimeter (Jasco, Tokyo, Japan) with a Peltier thermo control element (Jasco), using teflon-sealed, polarimetrically checked quartz glass cuvettes (Hellma Analytics) with an optical path length of 1 mm and a volume of 300 μL. CD spectra were obtained between 195 nm and 250 nm, using a wavelength increment of 1 nm, a scan speed of 10 nm/min and a response time of 5 s.

### Lipid II synthesis

Undecaprenyl phosphate (44.6 nmol, dried from an aceton solution), UDP-MurNAc-pentapeptide (crude extract from *Staphylococcus simulans* [28], 10 μL), Triton X-100 (0.5%), Tris-HCl (100 mM, pH 8.0), UDP-GlcNAc (0.67 mM), MgCl_2_ (6.7 mM) were mixed and finally membrane containing MraY or purified protein (0.01μg MraY and 0.01μg MurG) was added (total volume: 75 μL). The mixture was incubated at room temperature for a defined period (1 to 16 hours) and the reaction was quenched by adding 75 μL of butanol/6 M pyridine-HAc (2/1, v/v), pH 4.2. The mixture was vortexed and centrifuged briefly to induce phase separation. The upper layer was isolated and washed with 50 μl water. Eighteen μL of the upper butanol layer was dried in a desiccator. The residue was dissolved in CHCl_3_/CH_3_OH (1/1, v/v) and spotted on a silica gel thin layer chromatography (TLC) plate. The plate was developed in a solvent mixture of CHCl_3_, CH_3_OH, H_2_O, and NH_3_ (88:48:10:1). The lipids were subsequently visualized by iodide staining. Quantification of the intensity of the Lipid II spots was performed using Image J. The image was first converted to 8 bit gray scale and a java script (Nonuniform_Background_Removal.java) was used to enable the background correction plugin in Image J. The Lipid II spots were marked one by one with a rectangle of a fixed size and the total intensity inside each rectangle was measured as the intensity of the Lipid II spot.

### Synthesis of dansylated UDP-MurNAc-pentapeptide

**Fig 1:**
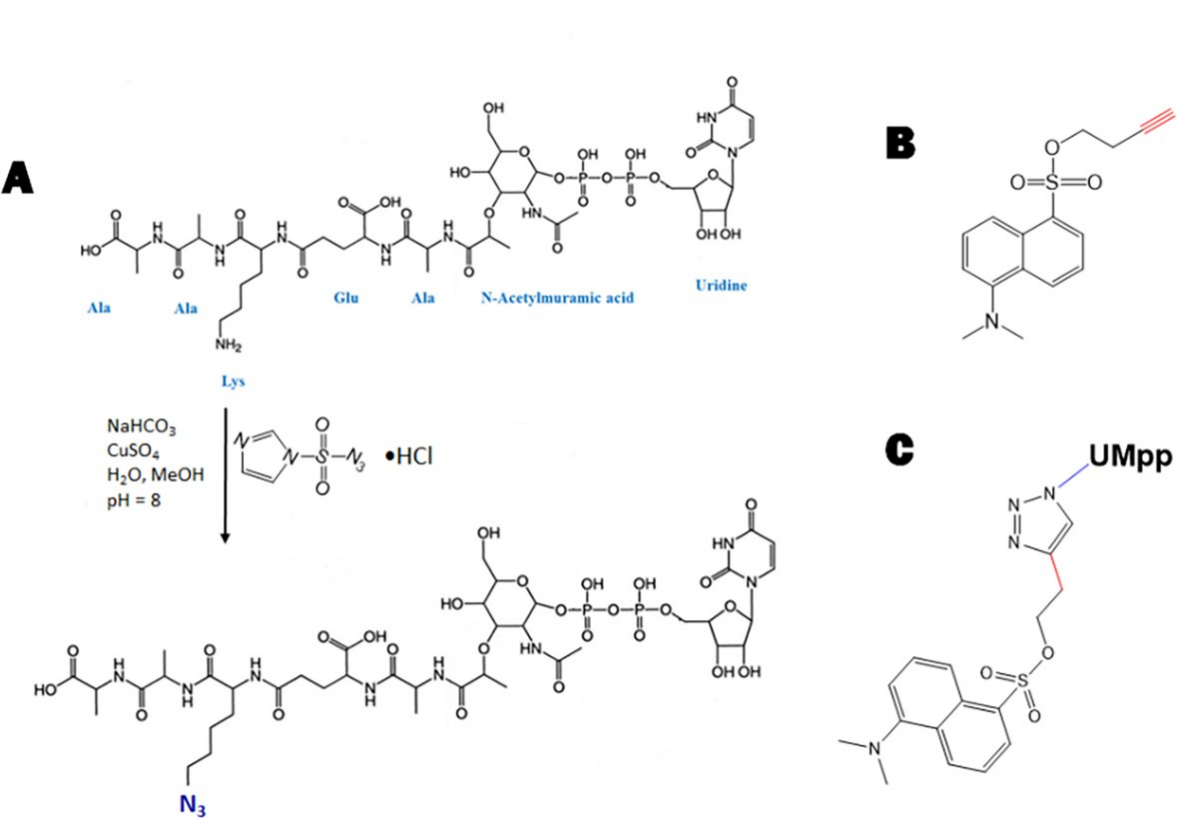
UDP-MurNAc-pentapeptide synthesis. (A) The principle of the reaction where the amine group of the lysine in UDP-MurNAc-pentapeptide was converted to azide through diazo transfer. (B) Structure of dansyl alkyne. (C) Structure of dansylated UDP-MurNAc-pentapeptide obtained through Cu(I) catalyzed azide-alkyne Huisgen cycloaddition.

UDP-*N*-acetylmuramyl-L-Ala-γ-D-Glu- L-Lys-D-Ala-D-Ala (UDP-MurNAc-pentapeptide, Fig 1) was dansylated through click chemistry. The amine group of the lysine in the peptide chain of UDP-Mpp was first converted to an azide through a diazotransfer reaction using imidazole-1-sulfonyl azide hydrochloride salt (ISA•HCl) as previously described [29] (Fig 1A). Dansyl alkyne was prepared as described in [30]. In short, dansyl chloride (0.37 mmol, 100 mg) was dissolved in dry DCM under water free conditions. 3-butyn-1-ol (0.44 mmol, 0.05 ml) and triethylamine (0.74 mmol, 0.1 ml) were added. The solution was stirred at room temperature overnight and then heated to 40°C for 4 h. After cooling the mixture was washed with 5% NaHCO_3_ solution. The solvent was removed yielding dansyl alkyne (Fig 1B). The UDP-MurNAc-pentapeptide azide was incubated with dansyl alkyne at room temperature overnight together with CuSO_4_ (1 mM) and sodium ascorbate (2 mM) to yield dansylated UDP-MurNAc- pentapeptide (Fig 1C). The purity of the compound is checked after HPLC purification (S1 Fig).

### Kinetics studies based on dansyl-Lipid I synthesis

Kinetics experiments were carried out following a previously reported protocol [20]. Total volumes of 50 μL consisting of 25 μM to 400 μM of C35-P, 15 μM to 100 μM DNS-UDP-Mpp, 200 mM Tris-HCl (pH 8.0), 0.5% Triton X-100, 50 mM MgCl_2_, 100 mM KCl were mixed in 96-well plates. MraY (10 nM) was added with thorough mixing to initiate the reaction. A fluorescence enhancement assay (n≥3, in high-throughput format) [16] was used to monitor Lipid I formation until a plateau value was obtained. This continuous fluorescence enhancement assay exploits the polarity-based fluorescence nature of dansyl group. The dansyl group, which fluoresces weakly (quantum yield < 0.1) in an aqueous environment [31], is brought into a non-polar hydrophobic environment (the detergent micelles) upon Lipid I synthesis. The emission shifts towards the blue (λ_ex_ = 346 nm, λ_em_ = 530 nm) and the quantum yield increases greatly. The fluorescence signal reaches a plateau when the reaction reaches equilibrium. Pure heptaprenyl phosphate (C35-P), an alternative to the natural lipid substrate (C55-P) of MraY, was either included to allow DNS-Lipid I synthesis or excluded in the reaction to obtain the baseline fluorescence signal of DNS-UDP-Mpp.On the assumption that equilibrium had been reached under these conditions and using the known equilibrium constant [32] of 0.25 for the phospho-transfer reaction, the fluorescence signal (in RFU, relative fluorescence unit) was converted into the concentration of DNS-Lipid I (702,239.3 ± 0.3% relative fluorescent units/μM) [20].

### Limited proteolysis by LysC

MraY was mixed with LysC protease (Lysyl Endopeptidase, Wako Laboratory Chemicals, Osaka, Japan) at a molar ratio of 1/100 (LysC/MraY) and incubated at 37˚C for 10 min. The reaction was quenched by adding 0.5% trifluoroacetic acid (TFA). The resulting mix was loaded on a 15% Bis-Tris protein gel [33] and visualized by silver staining [34].

## Results

### Comparison between detergent and SMA mediated MraY purification

**Fig 2:**
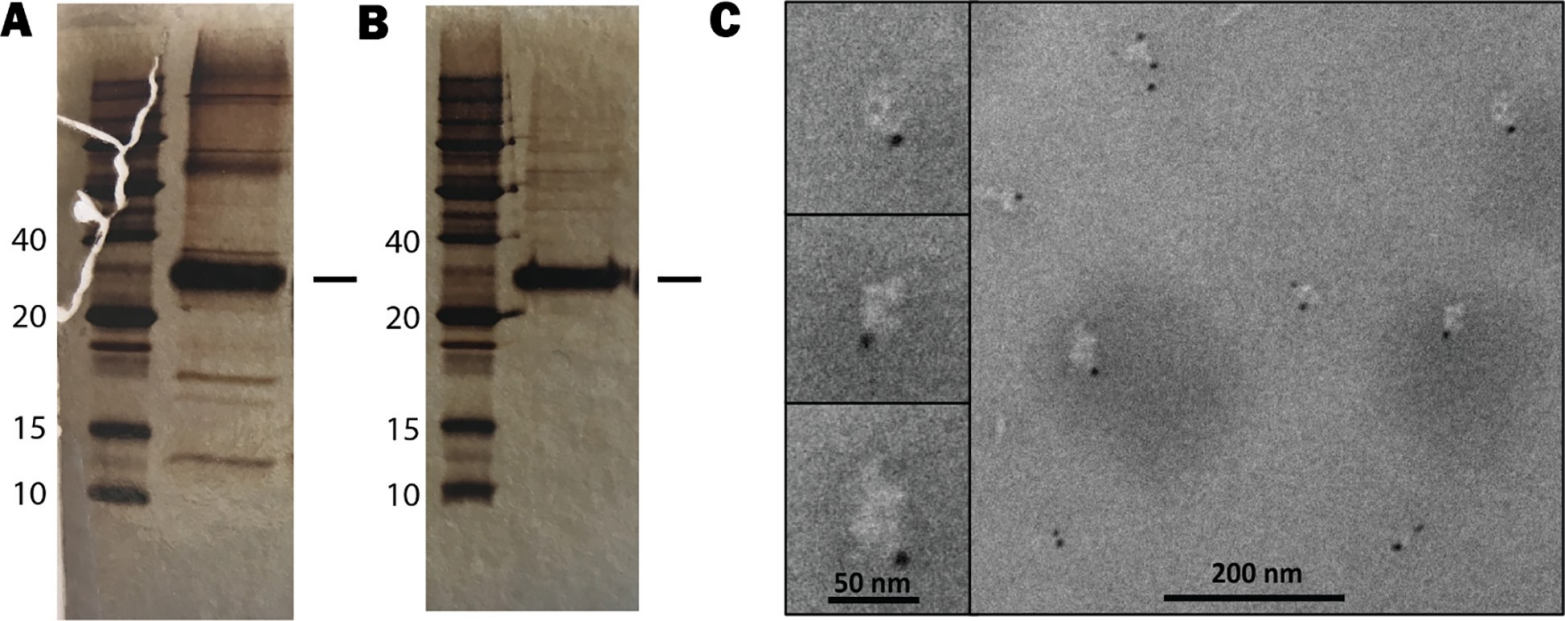
Recombinant MraY extracted (one-step) from *B*. *subtilis* using two different solubilization approaches. Purified MraY (indicated by a black stroke) solubilized by the detergent DDM (A) or solubilized by SMA (B). Protein bands were visualized by silver staining. (C) Transmission electron microscopy images of MraY in SMA-wrapped nanodiscs after nano-gold labeling. Three nanodiscs from the overview are shown in the enlarged images in the left panel (scale bar: 50 nm).

Extraction and purification of sufficient amounts of MraY with conventional methods have proven difficult. As an alternative approach to detergent solubilization, solubilization by SMA was first checked for its efficiency in obtaining pure protein in comparison with the DDM detergent system. Fig 2A and 2B show MraY (indicated with a black stroke, molecular weight of about 31 kDa) solubilized with DDM (DDM-MraY) and MraY solubilized with SMA (SMA-MraY) followed by a single purification step using a Ni^+^-NTA-agarose column. As depicted in Fig 2A and 2B, some impurities were present when DDM was used (46.8%), while solubilization by SMA yielded protein with apparently higher purity (93.9%). It should be noted that the DDM system did not always lead to a consistent yield of pure protein. Moreover, two solubilization steps of the membranes were usually required to achieve acceptable yields and at least two purification rounds from the Ni-NTA-agarose beads had to be carried out to yield sufficiently pure protein for further studies. Fig 2B shows the fraction that contain MraY solubilized by SMA. The purity of SMA-MraY is higher than DDM-MraY achieved with a single Ni-NTA agarose-based purification step. Since no membrane isolation was required prior to solubilization, the use of the SMA polymer to extract MraY makes the purification procedure much less cumbersome and less time consuming. Both methods yielded a comparable amount of pure protein: ~0.04 mg/L culture using SMA polymer, and ~0.03 mg/L culture using the DDM detergent system.

### Biophysical characterization of MraY in SMA-wrapped nanodiscs

To verify if MraY that was solubilized by SMA is indeed embedded in nanodiscs, we used Ni-NTA- nanogold particles of 5 nm to label the His_6_-tagged MraY. When examined using transmission electron microscopy (Fig 2C), the Ni-NTA-nanogold particles attached to the His_6_-tagged purified MraY were often observed on the edges of the nanodiscs, although some unbound particles were present most likely due to inefficient washing. The SMA-MraY nanodiscs have a somewhat irregular shape and display an average diameter of about 30 to 50 nm. This is larger than the commonly observed ~10 nm for different membrane proteins varying in size and shape [2, 4, 27]. It is conceivable that MraY may be present in the nanodiscs in a dimeric form since the size of the nanodiscs easily harbor an MraY dimer. This is supported by the observation that some of the nanodiscs are labeled by two nanogold particles, as shown in Fig 2C. In line with this, *Aquifex aeolicus* MraY has been crystallized as a dimer [21], and a more recent study reported that *B*. *subtilis* MraY is always present in dimeric form [19]. On the other hand, there have been reports about proteins larger in size than MraY being embedded in smaller SMA-wrapped nanodiscs [3, 5, 6, 35] and significant variations in size and shape of SMA-nanodiscs have been reported, irrespective of the size of the protein being embedded [1]. It is therefore more likely that the relatively large size and irregular shape of the particles is due to the formation of small aggregates of the SMA-wrapped nanodiscs.

**Fig 3:**
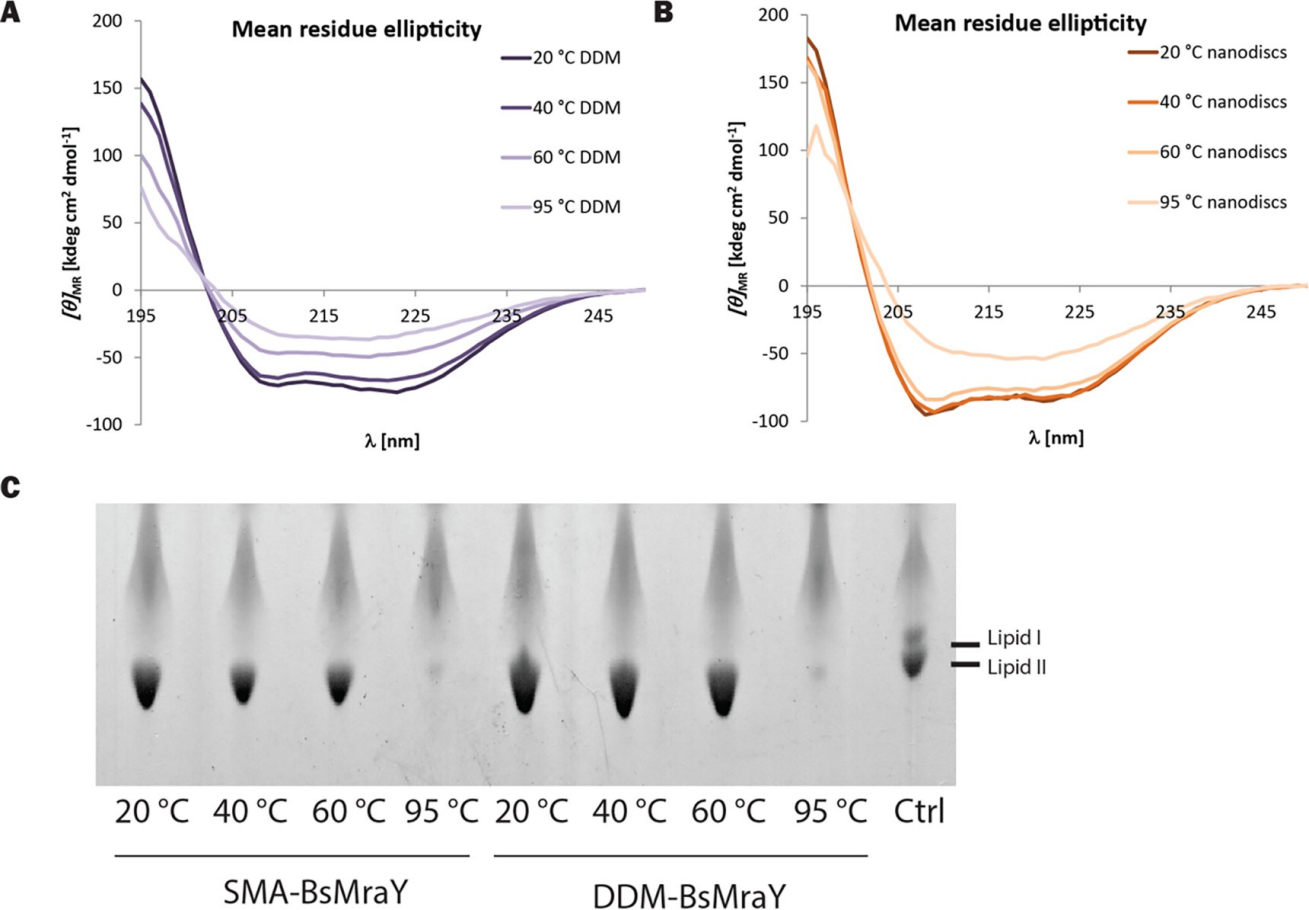
Thermal stability of purified MraY. (A-B) Normalized circular dichroism (CD) spectra of MraY purified in DDM micelles or SMA-wrapped nanodiscs are shown. Spectra were recorded at 4 different temperatures from 20°C to 95°C. Data are offset-corrected averages of 10 scans; (A) CD spectra of DDM- MraY; (B) CD spectra of SMA-MraY; (C) A representative TLC analysis of Lipid II synthesized from SMA-MraY or DDM- MraY incubated at 4 different temperatures prior to activity test at room temperature.

Further structural characterization of MraY in both DDM micelles and SMA-wrapped nanodiscs was achieved by circular dichroism (CD) spectroscopy (Fig 3A and 3B). Far-UV CD spectra of both preparations recorded at 20°C show minima at 208 and 222 nm that are characteristic of alpha-helical secondary structure, consistent with the recently-reported crystal structure of a homologous MraY [21]. However, the shape of the spectra obtained at room temperature display clear differences with the ratio of the minima at 208 and 222 nm changing from 0.90 for DDM-MraY to 1.13 for SMA-MraY, indicating an influence of the membrane-mimetic environment on MraY structure. More pronounced differences are revealed upon heating, with DDM-MraY losing typical alpha-helical features and exhibiting an overall decrease in signal intensity consistent with unfolding at 60°C. In contrast, the spectrum of SMA-MraY at 60°C strongly resembles that at 20°C, indicating conservation of the structural integrity. Further increase of the temperature to 95°C led to a similar thermal unfolding of SMA-MraY as for DDM-MraY, indicating that the melting temperature is higher in SMA-wrapped nanodiscs compared to detergent, as has also been reported for other membrane proteins [27, 36, 37].

Next, to see whether the thermal stability affects the enzyme activity, an MraY-MurG coupled Lipid II synthesis assay was performed using saturating amounts of undecaprenyl-phosphate. The two enzyme preparations were heated up to a defined temperature for one hour prior to the assay, which was performed for 16 h at room temperature. It was observed that protein heated up for one hour to 60°C in both cases remained active in Lipid II synthesis. Apparently in both cases the protein retained the ability to refold to its active form. A complete activity loss of MraY in both systems was only observed after the enzyme was heated at 95°C (A representative TLC image is shown: Fig 3C, lanes 4 and 8).

### Kinetics of the MraY-based Lipid II synthesis

**Fig 4:**
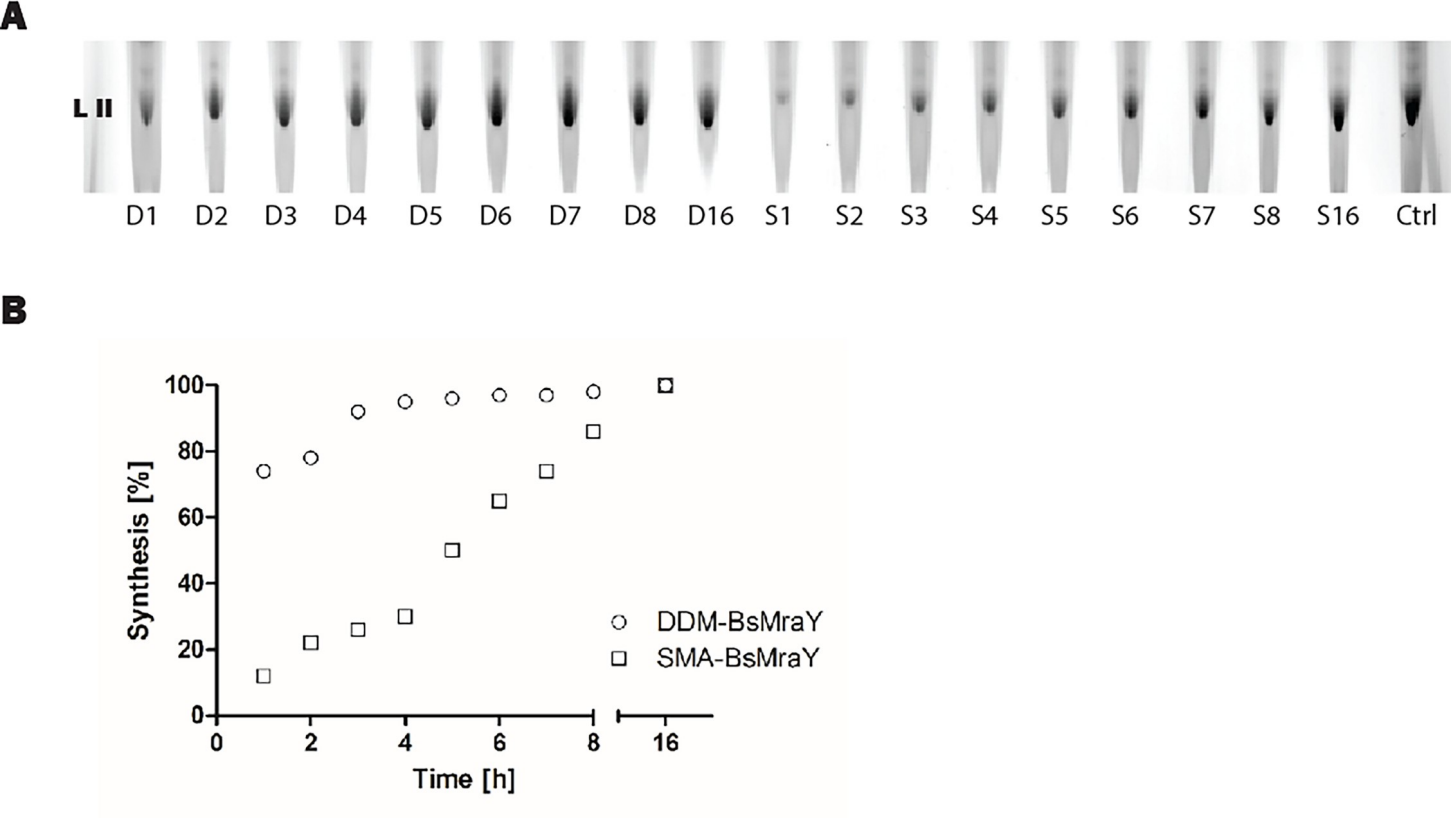
Kinetic analysis of Lipid II synthesis with MraY. (A) TLC analysis of Lipid II synthesis with either DDM-MraY (D1-16) or SMA-MraY (S1-16). The first spot from the bottom of the plate represents Lipid II (indicated as LII in the figure). (B) The intensity of Lipid II spots was analyzed by Image J and plotted against the incubation time. Reaction of 16 hours resulted in the most intensive Lipid II spot on the TLC plate for both enzyme preparations and were assigned as 100% activity recovery.

Although both enzyme preparations were shown to be active even after heat stress (previous section), it is possible that the environment does influence the kinetics of the 16-h Lipid II synthesis reaction. To check this, the reaction was followed in time by taking samples each hour (Fig 4A). The intensity of the Lipid II band from 16-h synthesis on TLC was similar to the control (synthesized with *Micrococcus flavus* membranes that contain naturally enhanced levels of MraY [38]). For better comparison, the intensity of the Lipid II spots was plotted against the time of incubation. Compared with the DDM-MraY, SMA-MraY showed a rather linear increase, with low amounts of Lipid II synthesized within the first 8 hours. Only at the end point (after 16 hours) the amounts of Lipid II that were synthesized were similar. DDM-MraY showed overall enhanced activity. A possible reason for this may be the accessibility of the protein to its lipophilic substrate. The substrate is solubilized in buffer with Triton X-100 and the apparent low activity in the early stages of incubation may reflect the time it takes for the substrate to partition from detergent micelles into the nanodiscs. Alternatively, it is possible that other factors may influence the activity of SMA-MraY.

### Lipid II synthesis is inhibited by free SMA

**Fig 5:**
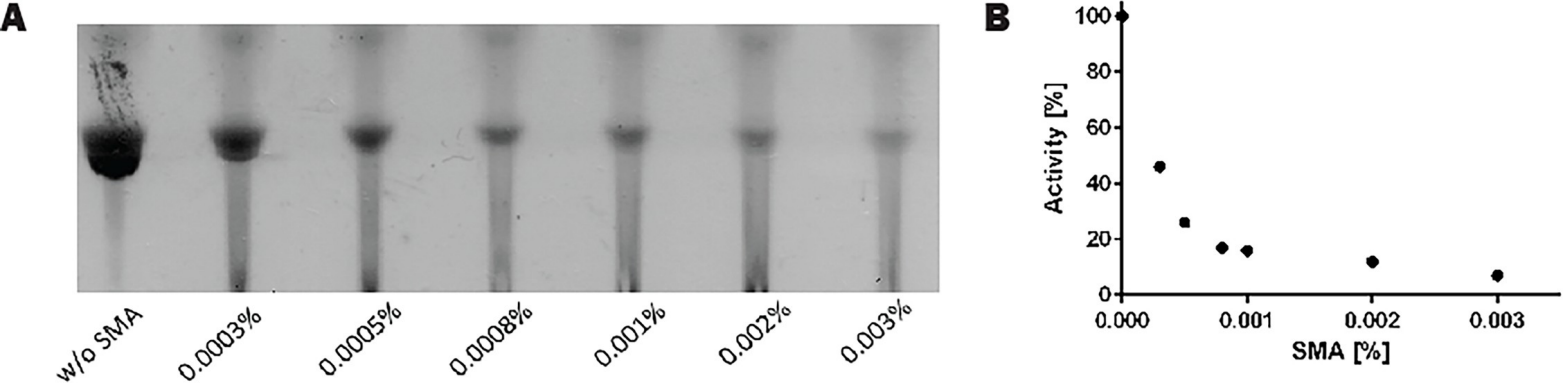
Effect of free SMA on Lipid II synthesis. (A) Lipid II synthesis with DDM-MraY (1 h incubation) visualized on TLC. First lane without adding free SMA. From second lane onwards increasing amount of free SMA was added; (B) Intensity of the spots on TLC shown in 5A was quantified using Image J and plotted against the concentration of SMA. Activity was set at 100% when no SMA was present. A dramatic drop of activity was observed as the SMA concentration increased.

It is plausible that during the Lipid II synthesis, SMA-wrapped nanodiscs are slowly disrupted by the detergent present in the system resulting in unbound SMA polymer. This free SMA polymer may have an effect on enzyme activity. We tested this by titrating SMA, from 0.0003% (w/v) to 0.003% (w/v), into the reaction mixture with DDM-MraY. Lipid II synthesis was visualized on a TLC plate and the result is shown in Fig 5A. The intensity of the Lipid II spot was analyzed by Image J and plotted against the concentration of SMA in Fig 5B. The intensity of Lipid II synthesized by DDM-MraY at 0% of SMA was set as 100% activity. Surprisingly, the amount of synthesized Lipid II dropped already by more than 50% when only 0.0003% of SMA polymer was added to the reaction mixture (Fig 5A and 5B). This suggests that added SMA polymer inhibits the enzyme activity. One possible explanation for such inhibition is that the presence of negatively charged SMA polymer may bind to the essential cofactor Mg^2+^, competing with its binding to the protein and thereby impairing its activity. To test this possibility, the activity of SMA-MraY was examined after addition of increasing concentrations of MgCl_2_, ranging from 7 to 50 mM.

**Fig 6:**
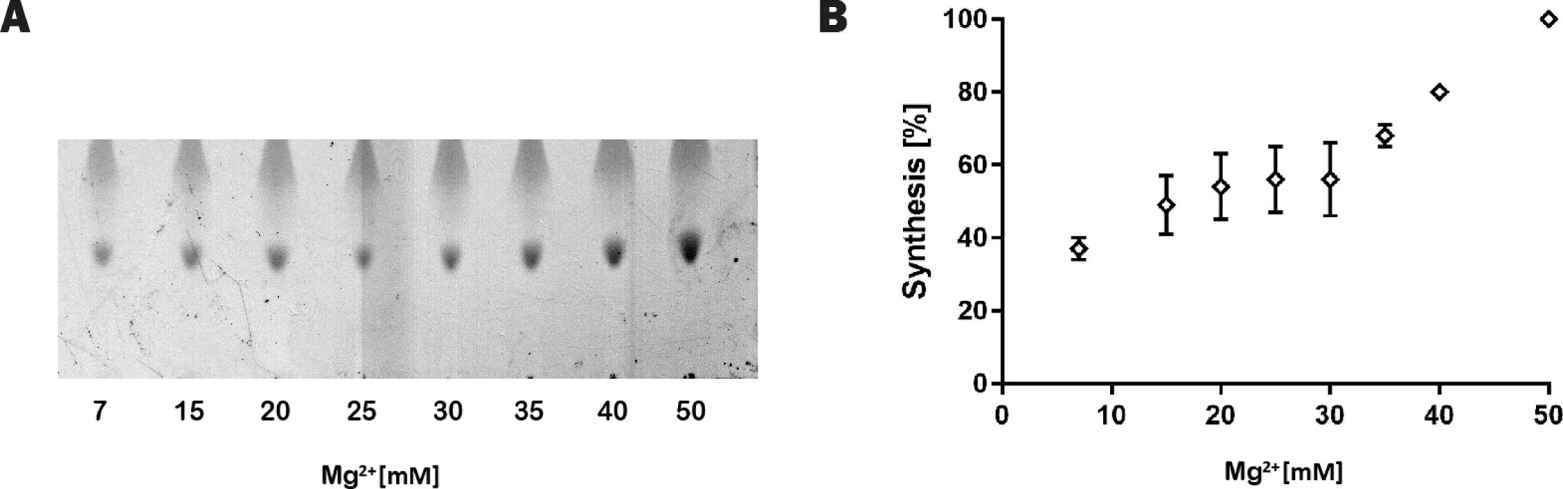
Effect of Mg^2+^ on Lipid II synthesis with SMA-MraY. (A) A representative TLC plate showing synthesized Lipid II (after 2 h incubation) spots with increasing amount of Mg^2+^; (B) Intensity of the Lipid II spots on TLC (n = 3) quantified by Image J and plotted against the concentration of Mg^2+^. Lipid II synthesis with 50 mM Mg^2+^ present was set as 100% activity.

The apparent activity of SMA-MraY was clearly improved as Mg^2+^ concentration increased (Fig 6A). Lipid II synthesis by SMA-MraY in the presence of 50 mM Mg^2+^ was comparable to the control (*Micrococcus flavos* membrane, 7 mM Mg^2+^) and was assigned as 100% activity recovery for clear interpretation of the data. The amount of Lipid II synthesis only slightly increased upon increasing the Mg^2+^ concentration up to 35 mM. Above this concentration, a steeper increase in the amount of synthesized Lipid II was observed (Fig 6B). When 50 mM of Mg^2+^ was included, the apparent activity was increased by three fold. This is considered nearly complete recovery of the activity of SMA-MraY, since the difference with DDM-MraY was about four fold (Fig 4B). This recovery cannot be explained simply by Mg^2+^ being no longer depleted by SMA, since 50 mM of Mg^2+^ is far more than what could be bound to SMA: in each reaction system about 2 mM maleic acid units is present and each maleic acid group carries one negative charge. The amount of Mg^2+^ is also higher than what is required for MraY activity [15, 39]. Moreover, it was previously reported that Mg^2+^ destabilizes SMA-wrapped nanodiscs [1, 7]. Taken together, it is plausible that Mg^2+^ addition together with the presence of detergent in the solution leads to destabilization of the nanodiscs, thereby allowing recovery of activity by increasing the accessibility of the protein to its substrate.

### Kinetics of the MraY catalysis

**Fig 7:**
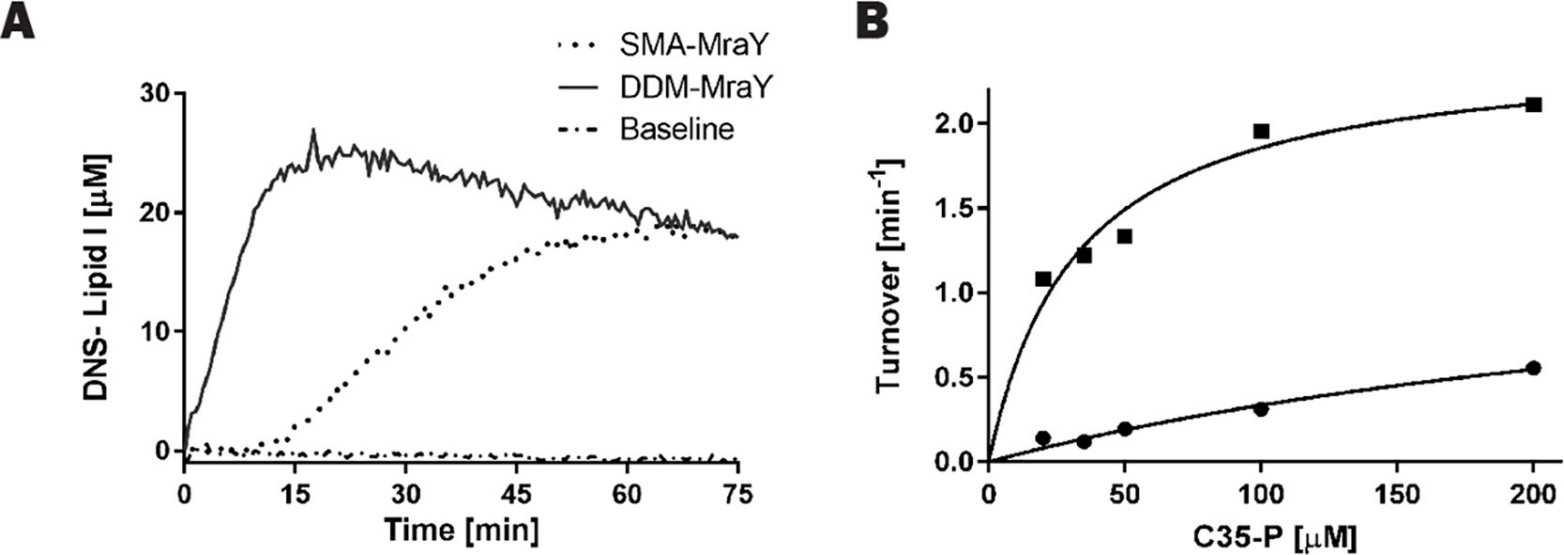
Kinetic analysis of Lipid I synthesis with MraY. (A) DNS-Lipid I production from 25 μM DNS- UDP-Mpp and 400 μM C35-P (in 0.5% Triton X-100) catalyzed by DDM-MraY (10 nM) or SMA-MraY (10 nM). Concentration of DNS-Lipid I was converted from the fluorescence signal as previously described (20). (B) Turnover rate of DNS-Lipid I production from 25 μM DNS-UDP-Mpp at several C35-P concentrations for DDM-MraY (■) and SMA-MraY (●).

The Lipid II synthesis assay involves another enzyme, MurG, in the reaction system. This makes the system too complex to determine the enzyme kinetics for MraY on its own or to test which component of the reaction affects the activity of the pure MraY. A fluorescence enhancement assay based on DNS-Lipid I synthesis was therefore previously developed [40]. DNS-Lipid I synthesis showed different reaction rates, as the equilibrium was reached within 20 min (Fig 7), compared with the TLC-based Lipid II synthesis (Fig 4). The difference in kinetics could be due to the exclusion of MurG as this enzyme uses Lipid I as its substrate and drives the synthesis towards completion resulting in at least one of the two substrates of MraY dropping in concentration.

This DNS-Lipid I synthesis assay was used as a standard assay to obtain kinetics data of MraY for both substrates (UDP-Mpp and C35-P) as was also described in our previous study [20]. As shown in Fig 7A, formation of DNS-Lipid I was monitored for SMA-MraY and DDM-MraY under identical conditions. While rapid formation of DNS-Lipid I was seen for DDM-MraY, SMA-MraY only began to show activity at least 10 minutes after initiation of the reaction.

In general, SMA-MraY catalyzes a slower synthesis of DNS-Lipid I than DDM-MraY does, similar to what was observed in the Lipid II synthesis assay. The flatter slope between 10 and 45 minutes indicates a lower turnover rate. In Fig 7B the maximum rate of production of DNS-Lipid I from 25 μM UDP-Mpp is shown as a function of C35-P concentration for DDM-MraY and SMA-MraY, respectively. The apparent K_m_ of C35-P (350 μM) for SMA-MraY is more than one order of magnitude higher than that for DDM-MraY (30 μM) at 25 μM UDP-Mpp. When we assume that SMA does not affect the Km or Ks values for UDP-Mpp, the hydrophilic substrate of MraY, tthe apparent K_m_ for C35-P can be estimated using the kinetics values previously reported in [20], resulting in 1.56 mM. This high Km of C35P and the low reaction rate at the initial stage of SMA-MraY catalyzed DNS-Lipid I synthesis indicate that C35-P solubilized in detergent micelles seems not to have straightforward access to the enzyme embedded in the SMA-wrapped nanodiscs.

**Fig 8:**
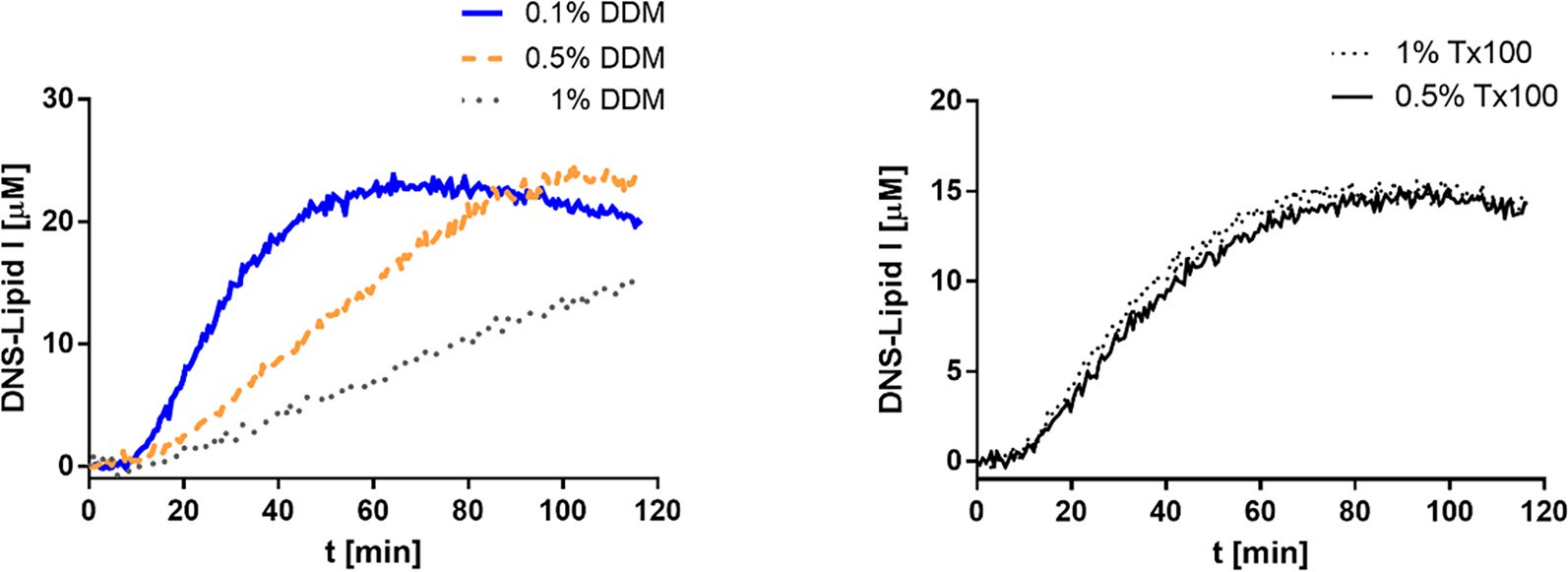
The influence of detergent on SMA-MraY activity. (A) DDM concentrations from 0.1% to 1% were used in DNS-Lipid I synthesis. The reaction was followed for up to two hours and it reached equilibrium faster when 0.1% DDM was used. (B) Triton X-100 at two different concentrations (0.5% and 1%) did not affect reaction rate as the time when the equilibrium was reached under these conditions was almost undistinguishable.

To determine whether detergent could be an influential factor for the activity tests, we also performed the reactions using DDM at three different concentrations to replace Triton X-100 in the Lipid I synthesis assay testing SMA-MraY. It was observed that increasing the DDM concentration reduced the reaction rate, while varying the concentration of Triton X-100 did not result in any change (Fig 8). These differences may be explained by the different properties of the two detergents. It was previously reported that DDM disrupts lipid bilayers at the onset of solubilization, and that also the micelle structure changes with increased detergent-to-lipid ratio [41]. In contrast, Triton X-100 does not disrupt the lipid bilayer structure at concentrations far beyond the CMC [41]. Taken together, DDM is likely to disrupt both the nanodiscs and the micelles more than Triton X-100, facilitating exchange of the substrate from the micelles into the nanodiscs and promoting accessibility of the SMA-MraY.

### Accessibility of MraY assessed by limited proteolysis

In the DNS-Lipid I synthesis assay with the SMA-wrapped nanodiscs system, the exchange of the lipid substrate and the enzyme between the nanodiscs and the micelles is essential for the synthesis. However this exchange does not occur immediately given the zero initial rate for about the first 10 min (Figs 7 and 8). Compared with the instant increase of fluorescence signal in the micelle system, this 10-min pause implies that the SMA-system needs sufficient time to initiate the reaction. Two scenarios may occur in principle:

(1) the lipid substrate partitions into the nanodiscs (2) the enzyme gets liberated from nanodiscs into detergent micelles. In order to get more insight into these possibilities, limited proteolysis [42] experiments on both DDM-MraY and SMA-MraY were carried out, which allow determination of differences in susceptibility towards proteases.

**Fig 9:**
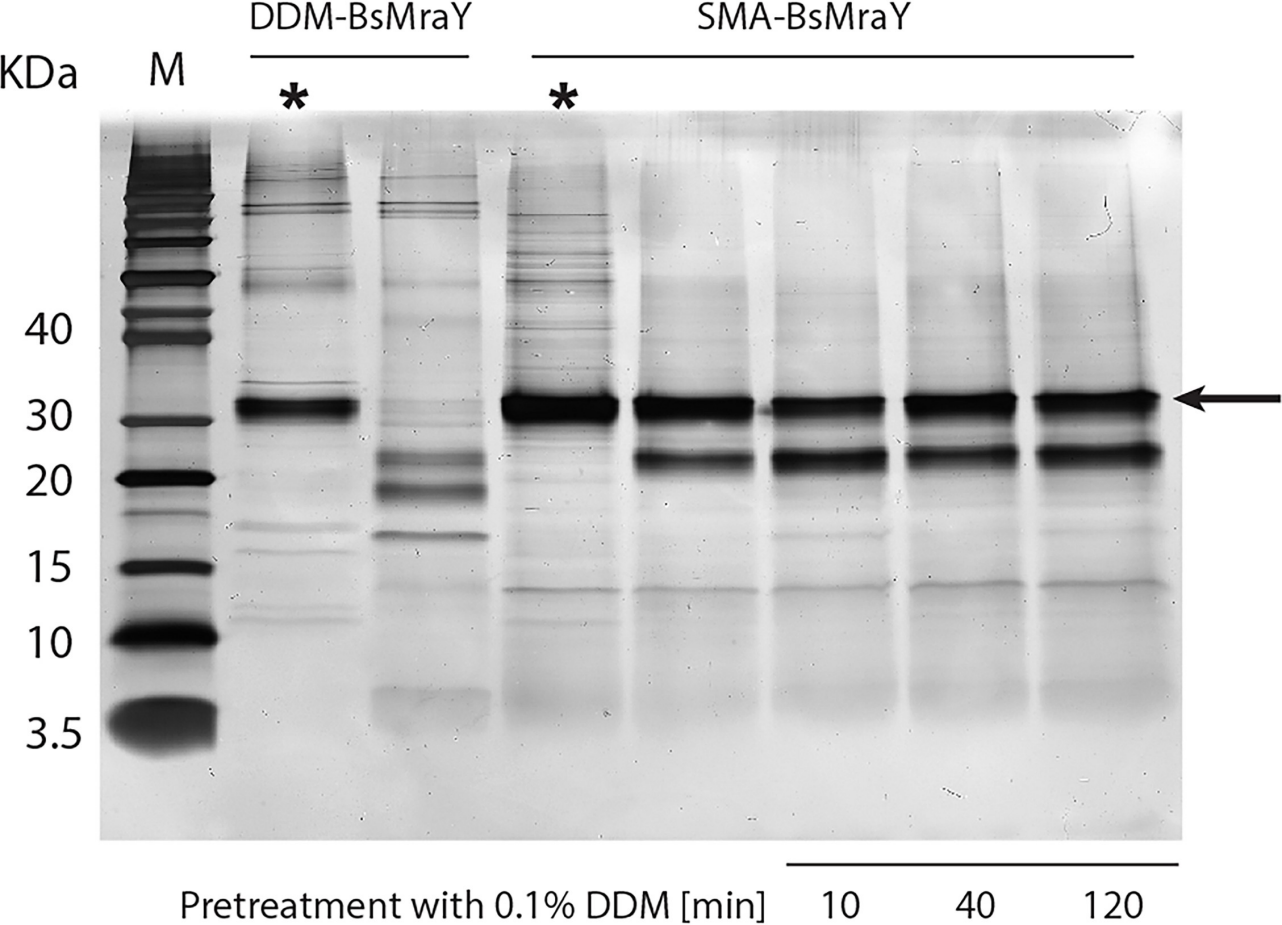
LysC treatment (37 ˚C, 10 min) of DDM-MraY and SMA-MraY. The influence of 0.1% DDM on the stability of SMA-MraY in the presence of LysC is shown after 10, 40 and 120 min incubation with the detergent at a final concentration of 0.1%. The lanes with the untreated protein are annotated with (*). M indicates the protein marker and the sizes (in kDa) are shown on the left side of the ladder. Intact MraY displays a molecular weight of about 31 kDa and is indicated by a black arrow.

LysC proteolysis led to virtually complete breakdown of DDM-MraY into several smaller fragments within 10 minutes (Fig 9). In contrast, SMA-MraY is much more resistant to proteolysis, while addition of 0.03% free SMA did not affect degradation of DDM-MraY caused by LysC (S2 Fig). In the absence of DDM detergent, only one major product (~ 25 kDa, probable cleavage at Lys32 in the second periplasmic loop) was formed after 10 minutes of incubation while the amount of intact protein (at ~ 31 kDa) was about 89% based on densitometric analysis. Pre-incubation of SMA-MraY with 0.1% DDM up to 2 hours resulted in the enzyme getting slightly more susceptible to proteolysis. On average 84% ± 3% of intact protein remained after LysC treatment. Again, one major product was formed, similar to that produced in the absence of DDM detergent. This observation shows that MraY remains embedded in the nanodiscs therefore the scenario of a complete dissolution of SMA-MraY into DDM micelles is invalid. This implies that the first scenario is more probable, and MraY embedded in the relatively rigid SMA-wrapped nanodiscs provides poor accessibility of the enzyme to its lipid substrate in the micelles, thereby causing the reduced apparent activity. These findings also indicate that SMA-wrapped nanodiscs can withstand detergent dissolution to a large extent.

Taken together, our results suggest that there is no complete liberation of the enzyme into detergent micelles. Instead, destabilization of the nanodiscs by detergent and accessing by the lipid substrate probably occur simultaneously during the Lipid I synthesis in vitro.

## Discussion

In the present study we successfully employed the recently developed SMA copolymer system to extract a membrane protein, the essential bacterial MraY, with its associated lipid components. Regarding the yield both methods were similar. Yet, a single-step SMA method produced MraY protein that was of higher purity (93.9%) than when using the DDM (46.8%). In addition to this, the SMA purification procedure provided significant advantages, which are the ease and the short turnaround time of the procedure, the thermostability of the protein, and the higher protease resistance.

The apparent activity of the SMA-MraY was reduced compared to the activity of the DDM-MraY. Our results show that the presence of free SMA polymer inhibits the activity of micellar MraY while additional Mg^2+^ rescued the activity of SMA-MraY. Given the heterogeneous nature of the reaction system, i.e. involvement of detergents, lipids, and ions, detailed kinetic studies with SMA-MraY were not carried out because of the poor performance of the enzyme under these conditions.

As outlined above in both Lipid II and DNS-Lipid I synthesis assays, the rate of the reactions catalyzed by SMA-MraY was slow compared to the reactions catalyzed by DDM-MraY. The reaction rate could be affected by other components in the synthesis assays, for example influence of Mg^2+^ on MurG activity. A steeper increase in the amount of synthesized Lipid II was observed with SMA-MraY beyond a concentration of 35 mM of Mg^2+^, while also a white sediment was observed in these mixtures with a relatively high Mg^2+^ concentration. This suggests that around this value, Mg^2+^ caused the unwanted free SMA to precipitate out of the solution and could no longer influence the assay. In addition it is possible that the SMA-wrapped nanodiscs may have precipitated, hence forming the white sediment. In this case, the nanodiscs most likely were strongly destabilized prior to precipitation, allowing the substrate from the micelles to partition into them.

It was previously reported that both substrates, UDP-MurNAc-pentapeptide and polyprenyl phosphate, have to bind to MraY concomitantly and closely near an essential histidine and the Mg^2+^ co-factor to enable the nucleophilic attack onto the pyrophosphate of UDP-MurNAc-pentapeptide. Thereby MraY may undergo a (slight) conformational change upon binding to the substrates [20, 22, 23]. The requirement that the polyprenyl phosphate, the lipid substrate of MraY, should bind in the hydrophobic groove of MraY formed by TM5, TM8, and TM9 indicates that the flexibility of the MraY transmembrane helices is important for its activity. Although previous studies have put much emphasis on the cytoplasmic side of MraY where the active sites of the enzyme are mostly located [13, 15, 16, 40, 43], our findings demonstrate that the binding of the lipid substrate to MraY is of great importance as well. Therefore, the apparent activity of MraY can be substantially affected when the binding to the isoprenyl phosphate is confronted with an extra barrier such as in the case of the SMA-wrapped nanodiscs.

During the10-minute lag time (Fig 7A) of the Lipid I synthesis by SMA-MraY, either the lipid substrate has to partition into the nanodiscs or the enzyme has to be liberated into the micelles in order to get to close proximity. Using limited proteolysis we could confirm that MraY largely stays in nanodiscs during the incubation time investigated (~2 hrs). While MraY in detergent is completely degraded by LysC treatment, only one major fragment of 25 kDa is formed. In any case, this implies that exchange of C35-P between detergent micelles and the lipid environment of MraY in nanodiscs is very slow, which is supported by the generally higher apparent Km of the SMA-MraY reaction. However, it was shown in a recent study using another SMA variant, that there is fast exchange of phospholipids (specifically DMPC and NBD-PE) between SMALPs [44]. This lipid exchange appeared to happen within seconds, presumably through fast collisional transfer [44]. However lipid-only empty SMALPs were investigated in this study, protein containing SMA-bound nanodiscs may behave differently. Another likely explanation for this discrepancy is that exchange is inhibited for C35-P because of its long hydrophobic tail.

We furthermore noted that the nanodiscs containing MraY are large (30–50 nm) and irregularly shaped. This tells us that even dimeric MraY (measuring 8 nm across) could easily be accommodated in these discs. Considering the unusual size and shape of the MraY containing nanodiscs we propose that the exchange of polyisoprene-phosphate from detergent may be further slowed down due to the formation of SMA-wrapped nanodisc aggregates that interfere with efficient uptake of the hydrophobic substrate in MraY. Finally, the extended polyisoprene-phosphate C35-P is longer than one membrane leaflet and its insertion into MraY- containing SMALPs may not be sufficient for activity, as it also needs to become properly accommodated into the binding pocket of MraY.

In general, SMA can be regarded as a promising alternative for extracting membrane proteins in their native lipid environment. However, when the method is applied to extract an enzyme (such as MraY) that requires binding of membrane-embedded (lipid) substrates to the transmembrane part of the protein, additional reconstitution into liposomes or detergent micelles may be needed. Our study shows that addition of a divalent cation, eg. Mg^2+^, is an effective approach to recover the apparent activity of the enzyme embedded in the SMA-wrapped nanodiscs, presumably by destabilizing the nanodiscs and thereby allowing the lipid substrate to be taken up more easily.

## Supporting information

S1 Fig DNS-UDP-MurNAc-pentapeptide on TLC. The left plate shows the fluorescence. The right plate is a reverted image. The right lane on each plate is the final purified compound after HPLC purification.
(TIF)

S2 Fig Free SMA does not inhibit LysC activity. DDM-MraY (lane 1) digestion by LysC at molar ratio protein:protease = 1:50 (lane 2) or 1:100 (lane 3). DDM-MraY digestion by LysC in presence of 0.03% free Xiran SZ30010 SMA polymer at molar ratio protein:protease = 1:50 (lane 4) or 1:100 (lane 5).
(TIF)
