# Correction: *Bacillus subtilis* MraY in detergent-free system of nanodiscs wrapped by styrene-maleic acid copolymers

**DOI:** 10.1371/journal.pone.0210135

**Published:** 2018-12-27

**Authors:** Yao Liu, Elisabete C. C. M. Moura, Jonas M. Dörr, Stefan Scheidelaar, Michal Heger, Maarten R. Egmond, J. Antoinette Killian, Tamimount Mohammadi, Eefjan Breukink

In the Author Contributions section, Tamimount Mohammadi (TM) should be listed as one of the persons who conceptualized and supervised the study.
