# Supplementary figures and images for: Bacillus subtilis MraY in detergent-free system of nanodiscs wrapped by styrene-maleic acid copolymers
Source: PLoS One. 2018 Nov 5;13(11):e0206692. doi: 10.1371/journal.pone.0206692 (PMC6218056; doi:10.1371/journal.pone.0206692)

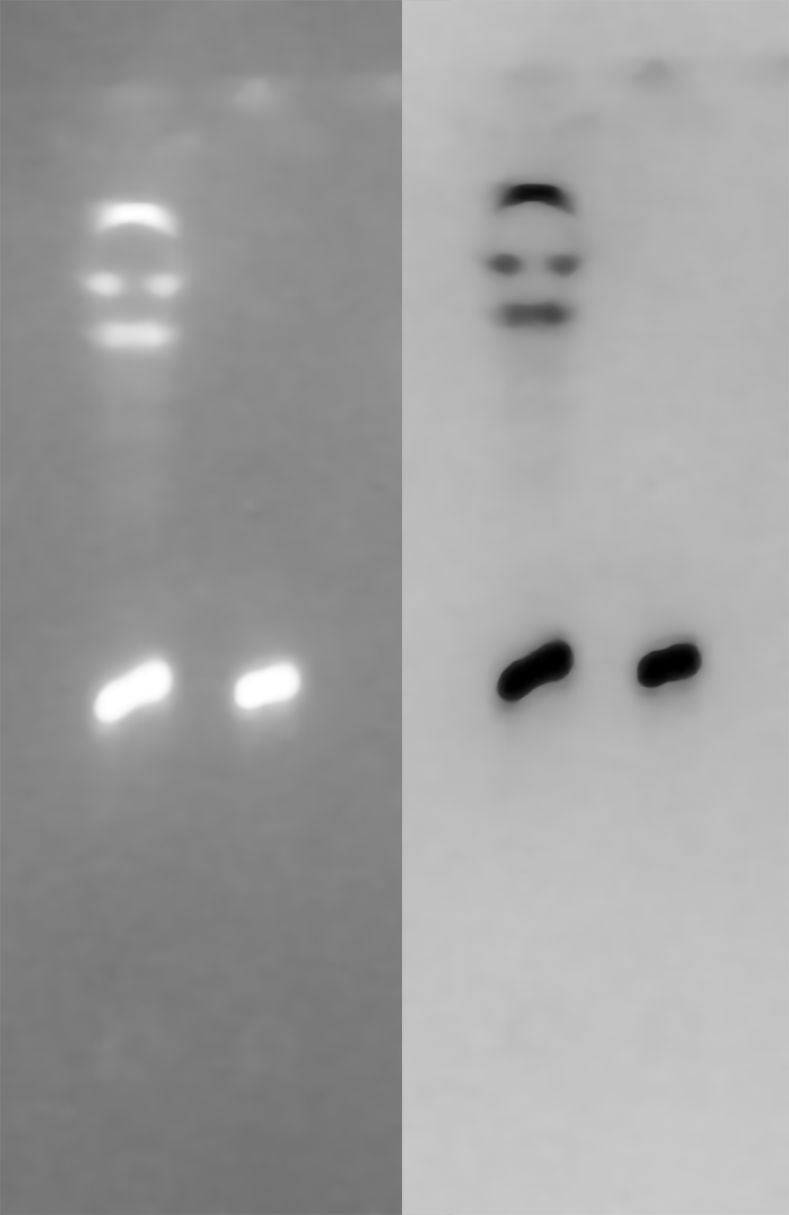

Supplement: S1 Fig — The left plate shows the fluorescence. The right plate is a reverted image. The right lane on each plate is the final purified compound after HPLC purification. (TIF) [file pone.0206692.s001.tif]

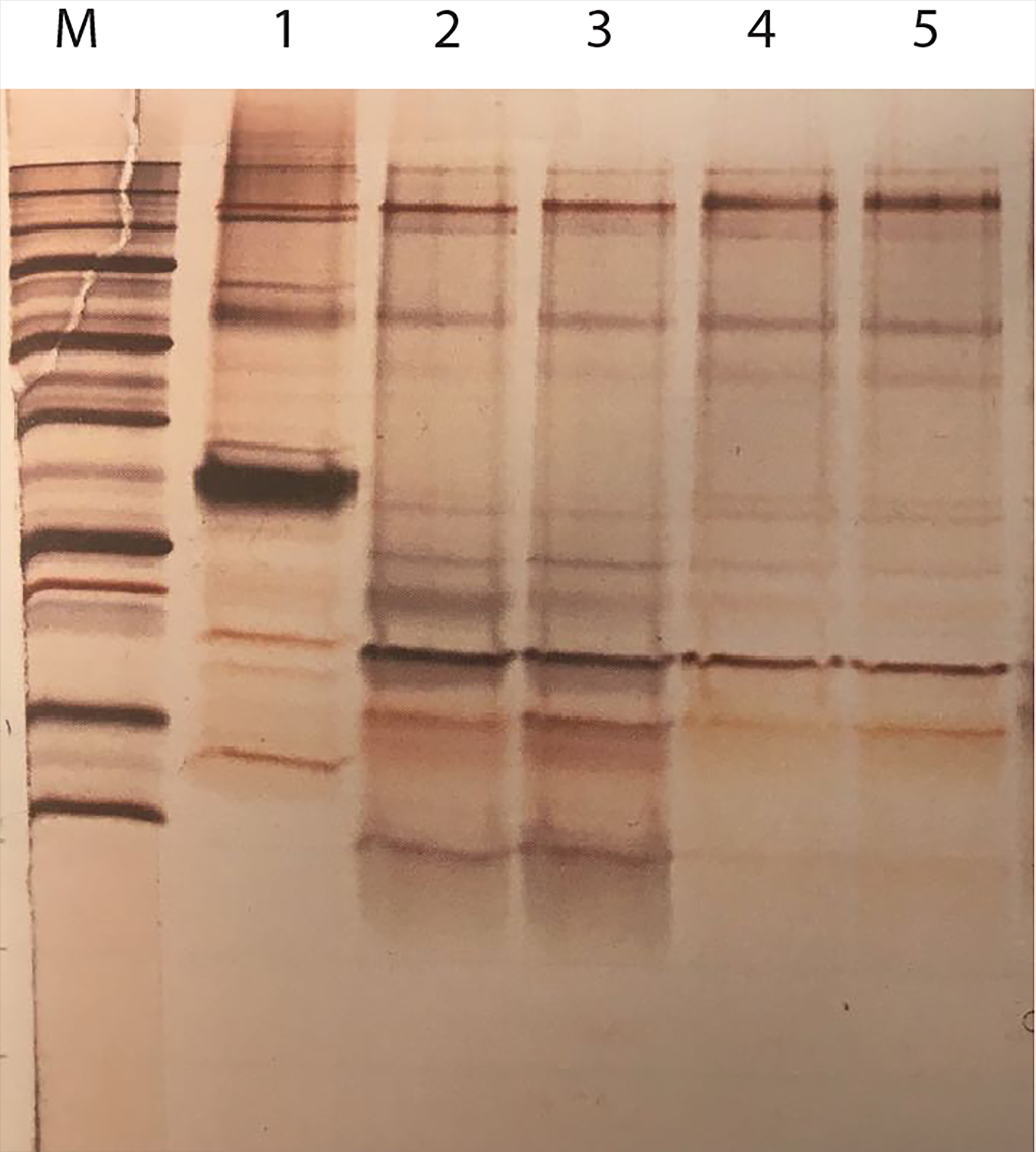

Supplement: S2 Fig — DDM-MraY (lane 1) digestion by LysC at molar ratio protein:protease = 1:50 (lane 2) or 1:100 (lane 3). DDM-MraY digestion by LysC in presence of 0.03% free Xiran SZ30010 SMA polymer at molar ratio protein:protease = 1:50 (lane 4) or 1:100 (lane 5). (TIF) [file pone.0206692.s002.tif]
